# Supplementary figures and images for: Oceanographic habitat and the coral microbiomes of urban-impacted reefs
Source: PeerJ. 2019 Sep 10;7:e7552. doi: 10.7717/peerj.7552 (PMC6743471; doi:10.7717/peerj.7552)

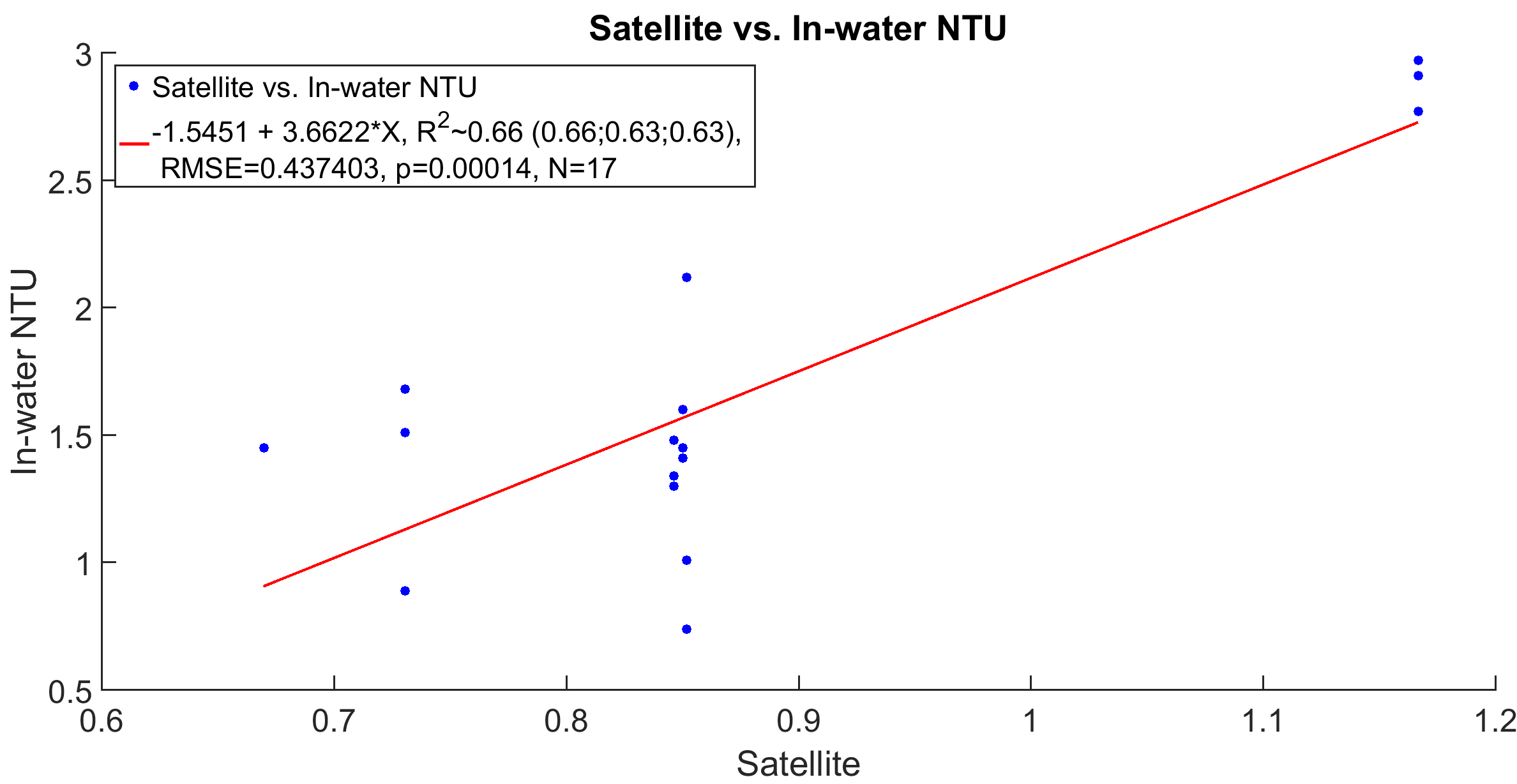

Supplement: Supplemental Information 1 — From from November 18, 2013—September 3, 2015, a cruise collected NTU data from Oakland Ridge reef, Barracuda reef, Pillars reef, and Emerald Reef. Only those in-water measurements meeting a sensitivity criterion (NTU > 0.5) were correlated to CI data to validate the model. NTU data was only used for validation of the model since the dates of tissue sampling did not correspond with the dates of ADCP data collections. [file peerj-07-7552-s001.png]

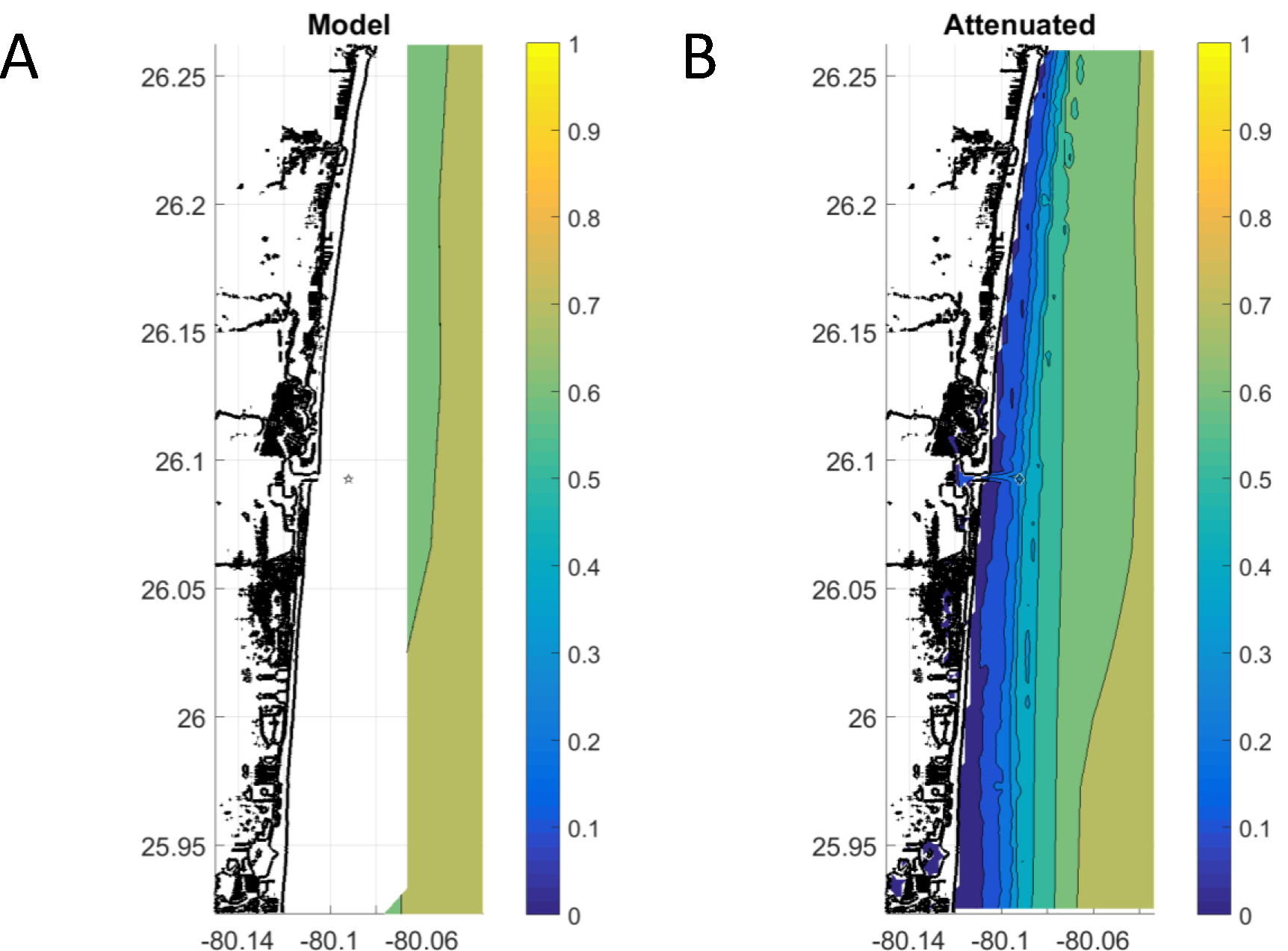

Supplement: Supplemental Information 2 — (A) For 2005–2015 for the northern portion of the sampling region. (B) Result of applying wave attenuation model using 10 m resolution bathymetry to the output of panel “A”. [file peerj-07-7552-s002.png]

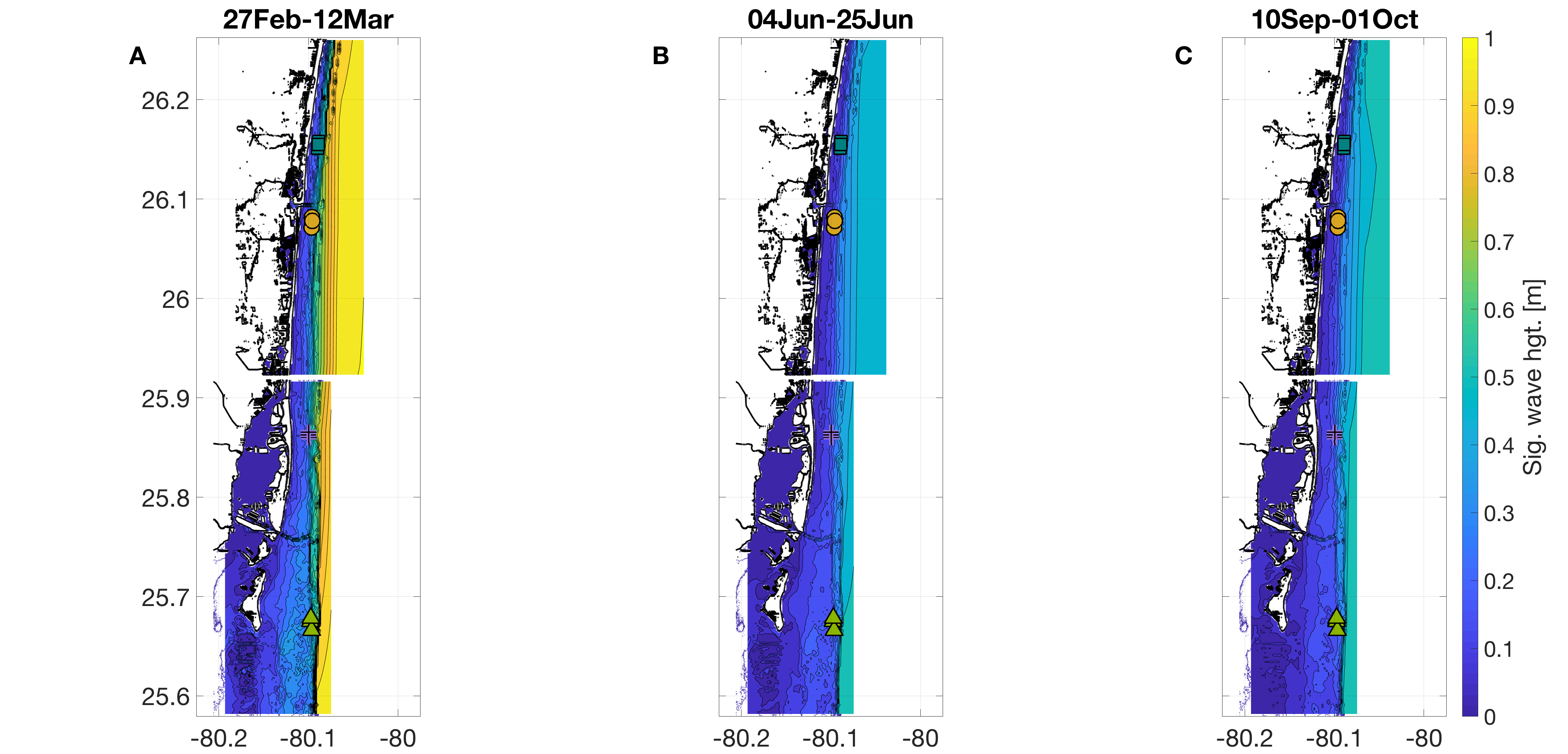

Supplement: Supplemental Information 3 — (A)March, (B) June, and (C) September. The 12 sampling sites are denoted by different shapes as follows: squares represent Oakland Ridge reef, circles represent Barracuda reef, crosses represent Pillars reef, and triangles represent Emerald Reef. [file peerj-07-7552-s003.png]

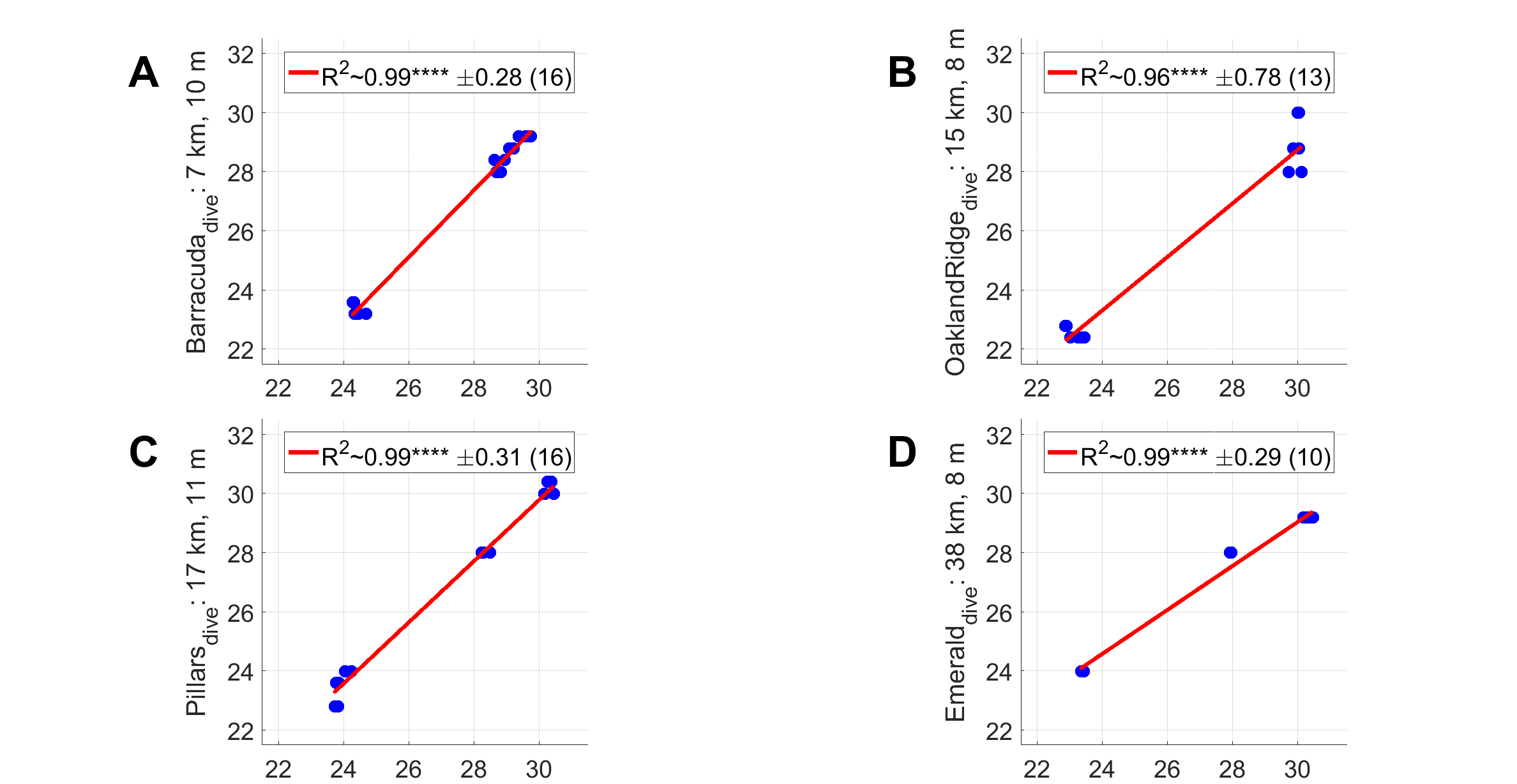

Supplement: Supplemental Information 4 — Reef location is indicated in the y-axis label of each regression plot, along with the distance from seven m mooring in km, and average sampling site depth for that reef. [file peerj-07-7552-s004.png]

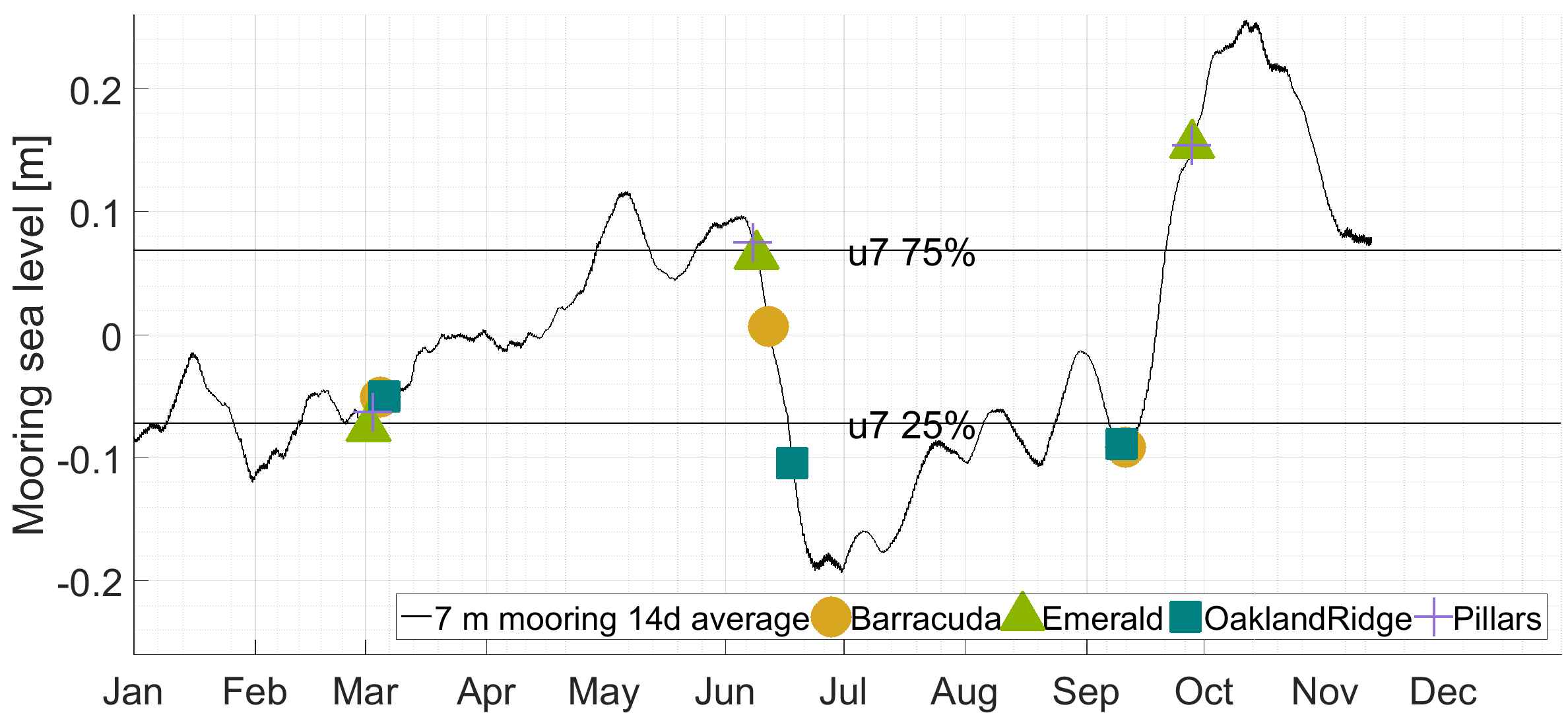

Supplement: Supplemental Information 5 — The FACE mooring at 7seven m depth (black line, time series; individual sample dates, colored stars). [file peerj-07-7552-s005.png]

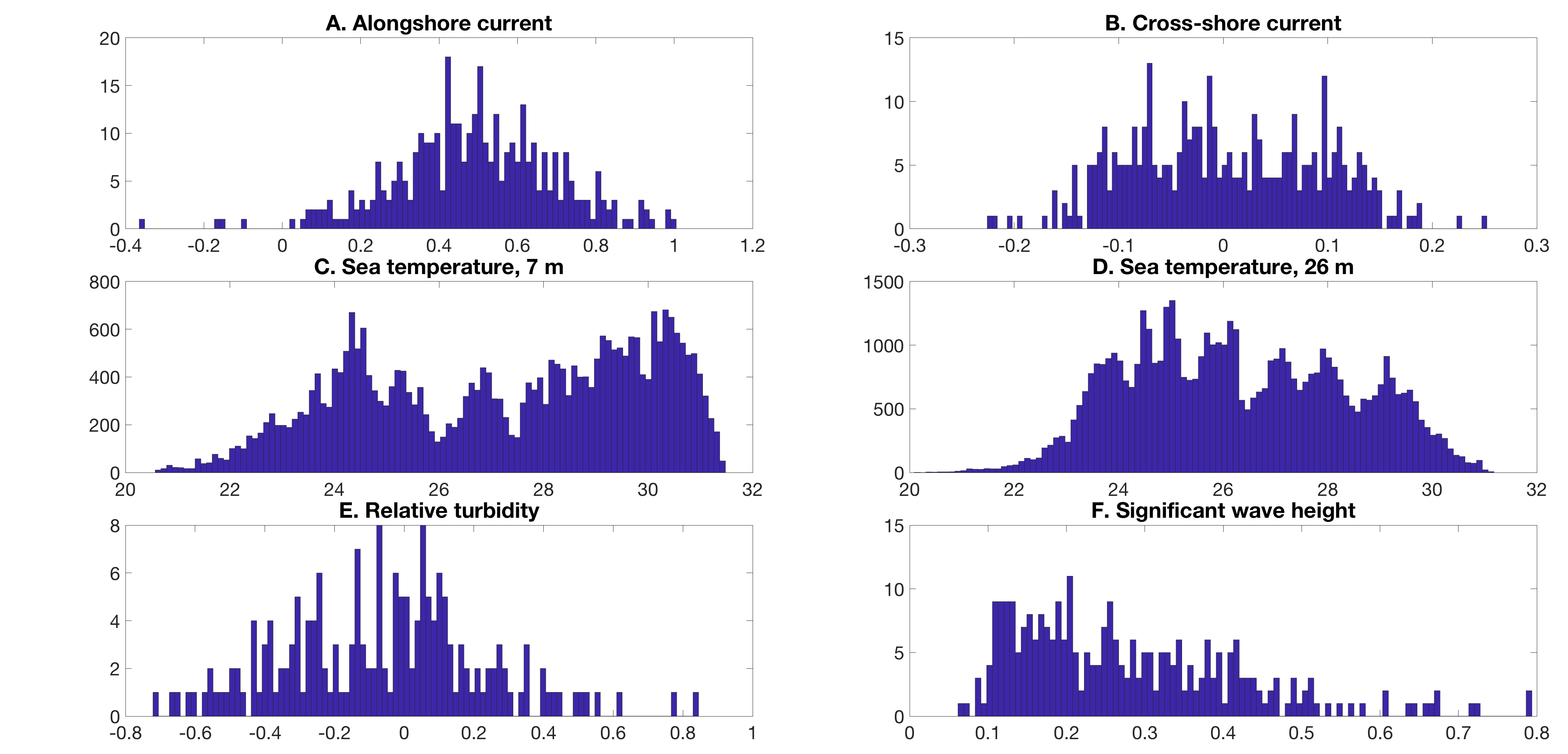

Supplement: Supplemental Information 6 — A) Alongshore currents (1 d−1, m s−1), (B) cross-shore currents (1 d−1, m s−1), (C) near-bottom sea temperature at seven m mooring (3 h−1,°C), (D) near-bottom sea temperature at 26 m mooring (3 h−1,°C), (E) relative turbidity (approximately 1 d−1), (F) significant wave height (1 d−1, m). Each distribution includes all available data from the year 2015. [file peerj-07-7552-s006.png]
